# List of Reviewers 2023

**DOI:** 10.1017/gmb.2023.19

**Published:** 2024-01-12

The Editorial Board of *Gut Microbiome* would like to thank the following for their contribution as peer reviewers in 2023:

Yogita Adlakha

Hanne Christine Bertram

Gabriela Bravo-Ruiseco

Emanuel E. Canfora

John Cryan

Jose F. Garcia-Mazcorro

Vo Van Giau

Simon Gray

Zubaidah Hasain

Devin B. Holman

Johannes R. Hov

Bruno P. Imbimbo

Marjukka Kolehmainen

Konstantinos Kormas

Aldons J. Lusis

Qin Ma

Isabel Moreno-Indias

Ravinder Nagpal

Paola Navarrete

Qin Xiang Ng

Viktoriya Nikolova

T. Requena

Jeffery Roach

Henning Seedorf

Yosra A. A. Soltan

Heidi Staudacher

Bruno Stefanon

Rupjyoti Talukdar

Zeneng Wang

Lucas Wauters

